# Acid Fruits

**Published:** 1891-02

**Authors:** 


### Acid Fruits.

The desire shown by the sick, and especially by those who are getting well, for acid fruits, as baked apples, cranberries, lemons, etc., should never be disregarded.

The important use the acids of fruits play in the body is a long story, so we can only insist upon regarding these "cravings" wherever found.

Sometimes the physician has good reasons for not wishing them given as the acid may neutralize, or decompose some remedial agent employed, but, as a rule, these fruits, properly prepared, may not only be given without injury, but with decided benefit.

So, whenever a sick person "craves" such things be sure to call the physician's attention to it, and ask if you can give them.

The question is often asked for the advantage of persons in health as well as the sick at what time in the day fruit should be eaten?

In tropical countries, where fruit is the chief article of food, the rule appears to be that the earlier in the day it is taken the better it is, and the later, the worse. In hot weather many wise people will eat none after noon, alleging that the digestion declines in power with the decline of the day, and the fruit instead of digesting, *decomposes*, owing to the presence of the saccharine matter. The objection to fruit and certain kinds of vegetable, late in the day, be the explanation what it may, is certainly justified by an ample experience.

When "taken for tea," especially if the person feels somewhat exhausted from the labors of the day, they do not appear to digest, but decompose, irretating the stomach and bowels until rejected during the process known as cholera morbus. Whenever this accurs do not put in upon that scape-goat the "liver," and take another dose of purgative medicine, but on yourself, for what you ate some hours before, and under what circumstances. If you use your experience another attack need not be feared for a long time. Many fruits and vegetables, such as melons and cucumbers, particularly if eaten immoderately under such circumstances, acquire the reputation of being "unhealthy" instead of which the eater is unwise. W.

